# Chronic Diarrhœa as It Occurred during and Since the Late War

**Published:** 1873-03

**Authors:** Alban S. Payne

**Affiliations:** Virginia


### SOUTHERN MEDICAL RECORD.

VOL. III.—MARCH, 1873.—NO. 3.

### PART I.

### ORIGINAL COMMUNICATIONS.

### CHRONIC DIARRHŒA AS IT OCCURRED DURING AND SINCE THE LATE WAR.

BY ALBAN S. PAYNE, M.D., OF VIRGINIA.

This disease became so common in the Piedmont region of Virginia during the year 1863, that it almost can be said to have prevailed as an epidemic. I shall, therefore, speak of it as "epidemic chronic diarrhœa," in contradistinction to the active and chronic forms of diarrhœa, as met with in *ante bellum* times. This chronic diarrhœa of war times, in its semiology, pathology and history, is, in my opinion, quite a different disease from the common chronic diarrhœa so truthfully described by the great Watson, and by his editor, my old and learned friend, Dr. D. Francis Condie. To establish this position, before I conclude this paper I will quote all Drs. Watson and Condie say of importance touching chronic diarrhœa as met with in private practice and public hospitals prior to the late great war in America.

A patient presents himself to me with diarrhœa. I at once ask the question, "Is your diarrhœa worse in the day-time or at night?" "It is worse after I go to bed at night." This is sufficient to satisfy me in my diagnosis of diarrhœa in its epidemic chronic form. And I then proceed to examine in reference to which variety of this disease his case belongs. If he says it is worse in the day-time, and does not bother him much in the night, I conclude his is a case of *ante bellum* diarrhœa, or at least a blending of epidemic chronic with *ante bellum* chronic diarrhœa, and is to be treated by rest in the horizontal position, low diet, or by clearing out the alimentary canal by a brisk bilious purge; and then by the administration of sedatives, or by a few grains of hydr. cum creta, combined with half a grain of gum opium and a quarter grain pulvis ipecac, to be given every four hours until his bowels are quieted; or a dose of castor oil is demanded for the cure. But I will let Dr. Watson and Dr. D. F. Condie speak for themselves, in their own beautiful and expressive language.

Dr. Watson says: "We frequently see this disorder supervene upon a debauch, in which case the *mixture* of various articles of food and of drink, each of which, in itself, might have been innocent—and the actual *quantity* of the ingesta—have occasioned the irritation and disturbance. But where there has been no intemperance in eating or drinking, some kinds of food are more likely than others, *cœteris paribus* to provoke diarrhœa. I do not speak of the idiosyncrasies, which show the truth of the old proverb, that what is one man's meat is another man's poison, and which cannot be reckoned upon beforehand; but I refer to the average of systems and stomachs. And among these indigestible and irritating substances we may place *raw vegetables* of many kinds, such as cucumbers and salads; sundry kinds of fruit, especially if they be hard, immature and acid—plums, melons, pineapples, nuts, and so forth. Mushrooms may be added to the list, even when they are cooked. *Putrid* food, or food which, in the more refined phraseology of gastronomers, is termed *high*, has the same effect upon some persons; and so, in a particular manner, have some kinds of *fish*—shell-fish, crabs and muscles, for instance, in this country; and in other countries, in the West Indies, there are several species of fish which are actually poisonous, and cannot be safely eaten at all. And similar disorder is frequently produced in children by any sort of food other than the natural sustenance furnished by the mother. The new kind of nutriment disagrees with them, and the very same thing is apt to occur in adult persons. An article of diet which is perfectly wholesome and digestible, and which the stomach bears well after a little habit, will sometimes cause griping and purging when taken for the first time. It is upon this principle that the diarrhœa to which Englishmen are subject upon first visiting the towns upon the continent is explained. I do not know that it is so, but I think it very likely that Frenchmen and Germans and Italians suffer in the same way when they first come to this country and adopt our habits and regimen.

"Another curious exciting cause is to be found in certain *mental emotions*, and especially the depressing passions, grief, and, above all, fear. A sudden panic will operate on the bowels of some persons as *surely* as a black dose, and much more *speedily*. Among the circumstances which *predispose* most persons to this kind of malady we may particularly specify *season*—the hot weather of summer and autumn. And it is probably consistent with the experience of most of you, that the atmosphere of the dissecting-room has a similar tendency.

"Now, this diarrhœa, from occasional irritation, produced by the presence of substances that offend the stomach, or bowels, will generally cease of itself. The purging is the natural way of getting rid of the irritant cause. We may *favor* the recovery by diluent drinks, and by making the patient abstain from all further use of food which is not perfectly easy of digestion; and we may often *accelerate* the recovery by sweeping out the alimentary canal by some safe purgative, and then soothing it by an opiate. Or, we may give the aperient and the anodyne together, and the one will not interfere with the operation of the other. A tablespoonful of castor oil, with six or eight minims of laudanum dropped upon it, or some fifteen grains to a scruple of powdered rhubarb with half as much of the *pulvis cretœ compositus cum opio.* By some such medication as this, emptying the bowels, and quieting them, the cure is generally accomplished with ease, and speedily: *tuto cito et jucunde.*

We sometimes, however, meet with cases in which diarrhœa *runs on;* the stools being composed of fecal matter in an unnaturally fluid state, and the precise condition on which this disposition to an over-loose state of the bowels depends escaping detection. If the disorder be slight, it will often yield to the astringent and bitter medicines. The infusion of cusparia with the tincture of cinnamon supplies a convenient formula. If it be more severe or obstinate, we have recourse to a chalk mixture, which neutralizes acidity, combined with catechu, which is a direct astringent of the tissues, and the laudanum, which calms irritation. And in extreme cases, the sulphate of copper has been found to have a powerful effect in restraining the flux. It is apt to gripe, and should be combined, therefore, with opium. A quarter of a grain of each, in a pill, given three or four times a day, I have frequently found successful when previous attempts to remove the diarrhœa had failed."

My learned and practical friend, Dr. D. Francis Condie, further says: "A much more effectual remedy is the acetate of lead combined with opium and ipecacuanha—one grain of the first, from a fourth to a half a grain of the second, and from a half a grain to a grain of the latter, combined in the form of a pill, or of a powder, mixed with a little simple syrup, may be given to an adult and repeated every three or four hours, according to circumstances. Diarrhœa in a chronic form is that which the practitioner will be the most frequently called upon to treat in the adult, and it, in general, requires, for its complete removal, a cautious and judicious course of treatment, persevered in for a long time. The slightest deviation from the strict diet and regimen required in each case will often very considerably protract the cure, while a too early abandonment of the appropriate remedies will frequently be quickly followed by a return of the worst symptoms of disease.

"In chronic diarrhœa there exists a morbid excitability of the intestinal canal, so that almost everything taken into the stomach as food or drink brings on quickly repeated discharges by stool, consisting of the ordinary secretions of the digestive tube more or less changed in character, mixed with half-digested aliment, and the looseness continues often unattended with griping or any other uneasy sensation, save those connected with the debility and emaciation produced by the interruption to the digestive and nutritive functions generally, which the rapid passage of the aliment through the bowels occasions.

There is no doubt that frequently the morbid excitability of the digestive canal is due to a chronic inflammation often follicular and attended with ulceration of some portion of its mucous membrane. When this is the case, we have repeated discharges by stool without apparently any exciting cause other than the morbid secretions of the liver, pancreas, or of the stomach and intestines themselves. The discharges are, in general, dark-colored and offensive, very fluid and small in quantity, and are often preceded and accompanied by griping pains more or less severe. There is very commonly some degree of nausea, and occasionally vomiting; the appetite for the food is generally destroyed, though in many cases it continues unimpaired. The patient becomes more and more emaciated and debilitated; his skin assumes a dirty, sallow hue, and a dry, harsh feel; the palms of the hands become hot and dry; the countenance has, in many cases, a dull, desponding expression; the features acquire considerable sharpness, and the eyes become shrunken and surrounded by a broad, leaden-colored ring. The abdomen is frequently flaccid, and exhibits no tenderness upon moderate pressure; occasionally, however, it becomes swollen and tympanitic, and now and then decidedly tender to the touch. We have known in cases of chronic diarrhœa an effusion of serum to appear within the peritoneal cavity, and to produce a very decided intumescence of the abdomen. In protracted cases the body exhales a peculiar sickly ordor, the tongue becomes of dark mahogany hue, and often, together with the parietes of the mouth, is covered with aphthæ. The pulse is usually small and feeble, often quick and frequent. Febrile symptoms are not generally present; in many cases, however, there is observed some degree of febrile excitement towards evening. Very protracted cases we have frequently known to be accompanied with well-marked hectic symptoms—more or less puffness of the face, an œdematous swelling of the extremities, very commonly occur in the course of the disease. The discharges by stool, while they are always fluid and vitiated, exhibit considerable variety in their appearance; most generally they are dark-colored, and exhale a rancid or fetid odor. Occasionally, however, they have a jelly-like consistence, and very little smell; at other times, they consist of a small quantity of dirty, yellow fluid, and when they contain solid matter, this will generally be found to consist of portions of half-digested aliment. All these changes in the character of the discharges may present themselves in the same case and often within a very short period.

"In protracted cases, the discharges would appear to acquire an acrid property, producing an erythematous inflammation of the verge of the anus, and often of the nates. The frequency of the stools varies very much in different cases, and at different periods in the same case. Occasionally the diarrhœa takes place only after the ingestion of food or drinks, or of particular kinds of food, and the discharges from the bowels continue to recur at short intervals until the offending matters are got rid of; in many cases repeated stools occur in the course of the day, whether food is taken or not, and are suspended during the night; in other instances, the evacuations from the bowels often cease for a day, or even longer, and then return, and for a short period with increased frequency. The duration of chronic diarrhœa is very various; unless arrested by a proper course of treatment—its spontaneous cessation being a thing of very rare occurrence—it will run on for weeks, often for months, and the patient finally sinks from extreme exhaustion. Occasionally perforation of the intestines occurs from ulceration or softening, and the fatal event is preceded by peritonitis.

"The causes of chronic diarrhœa are the same as the acute or simple form of the disease. Frequent attacks within a short period of an ordinary bowel complaint will very commonly induce a chronic affection. Improper articles of diet and acescent drinks, habitually indulged in; exposure to cold, and, at the same time, humid atmosphere; the abuse of purgatives and intemperate habits generally, are among the most common causes of chronic diarrhœa. It is an affection much more readily induced in those of a lax, feeble, excitable and broken-down constitution, than in those of an opposite condition. The state of the intestinal tube in those who have fallen victims to the disease is very various. In some cases the mucous coat, particularly of the large intestines, is somewhat thickened, spongy and pale—in others its anatomical characters are entirely changed, large portions of it presenting a smooth, glassy, mottled appearance, as though its surface had been covered with a thin coating of dirty varnish. Occasionally large patches of the mucous membrane of the colon or rectum are of a dark mahogany, or of a slate color. The traces of follicular inflammation, or of ulceration more or less extensive, are not unfrequently met with, especially in the ileum or colon.

"Dr. Stokes notices a form of chronic diarrhœa as of common occurrence, dependent upon ulcers situated close to the verge of the anus; these ulcers occur chiefly in persons of broken-down constitution, and those who have taken a great deal of mercury; we have repeatedly observed them also in individuals who have been in the habit of using, almost daily, the various pills composed chiefly of aloes, soap and scammony, or gamboge, vast quantities of which are vended in the United States as a popular remedy for almost every ailment. The ulcers situated just within the anus produce irritation in the colon, tenesmus, griping, frequent discharges by stool, and most commonly during the straining a little blood is passed. The presence of ulcers may be at once detected by an examination of the rectum, which examination, as Dr. Stokes very correctly remarks, should invariably be made in all cases where the diarrhœa has been of long standing and has resisted a variety of treatment; where it is attended with tenesmus, and a desire of sitting on the night-chair after the stool has been passed; and finally, where the patient's health does not seem to be so much affected as it naturally would be from long-continued disease of a large portion of the great intestine. In the treatment of chronic diarrhœa, our leading indications are to control the morbid irritability or excitability of the intestinal mucous membrane, and restore it as quickly as possible to its healthy condition and functions. To effect these objects is not always, however, a very easy task, and always demands considerable judgment on the part of the practitioner, and considerable patience and an implicit obedience on the part of the patient to the medicinal directions and dietetic rules laid down. The first and all-important consideration is that of diet—so that while the patient is supplied with aliment calculated for his support, as little irritation as possible of the intestines shall be excited by it. The food taken by an individual laboring under chronic diarrhœa should be easy of digestion, of the mildest quality, and such as leaves, after undergoing digestion, but a small quantity of excrementitious matter; and even of such food, but a small portion should be taken at a time. Rice is probably the best article of diet in the generality of cases of chronic diarrhœa; when well boiled, with the addition of a little salt, while it is sufficiently nourishing, it is extremely mild and unirritating, by no means difficult of digestion, and scarcely affords any excrementitious matter to be transmitted along the intestines. It may generally be eaten mixed with a very moderate quantity of plain beef or mutton broth; plain meat broths, prepared with the addition of a large amount of rice, will often furnish a very suitable food in chronic diarrhœa, and are to many stomachs more palatable; rice also boiled with milk, and sweetened, but not too heavily, with the best loaf sugar, or fresh milk thickened with rice flour, may be occasionally given. Should either preparation, however, be found to disagree with the patient, or to augment or keep up the diarrhœa, it should be at once relinquished. We have indeed, in numerous instances, found plain broths, when well prepared, or the juice of roasted meats, with a portion of stale bread or cracker broken into it, agree better than any preparation of rice. Tapioca, sago or arrow-root we have seldom found an appropriate aliment for persons laboring under the chronic form of diarrhœa. As soon as it can be borne—and this can only be ascertained by a cautious trial—a small portion of tender chicken, turkey or mutton, plainly boiled or roasted, may be eaten with rice. Pure water toast or rice water, taken cold and in very moderate quantities at a time, should be the only drink allowed.

"Next, or more properly, perhaps, equal in importance to well regulated diet, is an attention to the clothing and regimen of the patient. Individuals affected with chronic diarrhœa are particularly susceptible to the influence of cold and damp atmosphere—a slight exposure to which will often increase their disease, or, when we have succeeded in diminishing the frequency of the discharges, cause them to return as before. It is essential, therefore, that, independent of cautiously avoiding every species of exposure, the patient should be suitably lodged and clothed. The chamber he occupies at night, as well as during the day, should be dry, of a comfortable and equable temperature, perfectly clean and well ventilated. His clothing should be adapted to the season and the state of the weather; flannel next to the skin should always be worn; a belt of flannel around the abdomen, or enveloping this part with a flannel roller, nicely adjusted and renewed daily, will always be found advantageous. In obstinate and protracted cases the removal of the patient from a cold, damp and changeable, to a more equable, warmer and drier climate, whenever practicable, is a measure from which the very best results are to be anticipated; it has, in numerous instances, been known to effect a speedy cure, when all other means have failed.

"In regard to exercise, even the feeblest kind, whether passive or active, cannot sometimes be undertaken, from the frequent and pressing calls to evacuate the bowels, which occasionally are found to be excited by motion of every kind. In other cases, short walks in the open air in suitable weather, or a gentle ride in an open carriage, or sailing in a boat, are advantageous, and should be repeated daily, if the patient's strength will admit of it. The warm bath, followed by a brisk friction of the surface, is a remedy from which the best effects are to be anticipated in most cases of chronic diarrhœa; it may be repeated daily, the temperature of the water being carefully graduated by the condition of the patient's surface: when this is dry and warm, a tepid bath should be preferred—but if the surface is cool, or its heat is not well sustained, the water should be decidedly warm. The temperature of the bath should never be so low as to cause the patient, when immersed in it, the slightest sensation of chilliness on the one hand; nor so high on the other, nor the continuance in it so long, as to produce profuse perspiration.

"Blisters to the abdomen will, we apprehend, be found more generally advantageous than leeches; we have found them to produce a speedy, marked and prompt amelioration in the prominent symptoms of the case.

"The principal internal remedies from which any effects are to be anticipated are opiates and astringents.

"We have not derived the same advantage in cases of chronic diarrhæa from the salts of morphium as from the opium itself. The vegetable astringents most deserving of attention are the catechu, kino, galls, logwood, blackberry root, and the root of the geranium maculatum. The first may be given in the form of the infus. catechu comp.

"Of the mineral astringents, we know of none superior to the acetate of lead; in the dose of one grain, combined with a quarter of a grain of opium and the same quantity of ipecacuanha, repeated every three hours, it will, in a large number of cases, promptly arrest the disease. The proto-carbonate of iron, the tincture of the chloride of iron, and the solution of the persesquinitrate of iron, we have repeatedly employed, and, in cases of long standing, have found them, especially the two first, very efficacious. They are particularly well adapted to protracted cases of the disease, attended with great prostration, and more or less infiltration of the subcutaneous cellular tissue. The balsam copaiba and spirits of turpentine are among the remedies from which, in numerous cases of chronic diarrhœa, the very best effects may be anticipated. A variety of other remedies are recommended by different writers, the efficacy of which is highly extolled. The most prominent are *Hope's Mixture*, which is a mixture of nitrous acid, camphor water and laudanum—the nux vomica and its active principle—the ferro-cyanuret of iron, the nitrate of silver, and the resinous extract of the *artemisea vulgaris*. Of the effects of these, we have had no experience."

This is a beautiful as well as most truthful delineation of the causation, semiology, pathology and treatment of the *ante bellum* type of chronic diarrhœa, which usually followed closely upon the heels of an acute attack of diarrhœa. This picture of this disease was drawn by master minds, and, I admit, perfectly exhaustive. But unfortunately, the picture so ably drawn by these Michael Angelos of our profession only partially touches the causation of the disease of which I write, and does not touch at all either its semiology or its pathological condition. After this rich display of choice and classical language, mine must needs be a nude and roughly-drawn picture; but, nevertheless, this is "epidemic chronic diarrhœa."

The disease usually commences and is always worse at night, after being in bed some few hours. His pulse is slow, weak, soft; his tongue commonly clean, slightly more dry than is customary in sound health, with an appearance of being glazed down its middle; edges redder than usual, with slight elongation of the papillæ; his skin cool and moist, giving to the hand somewhat the feel of the cadaver; his mind clear and unclouded; his thirst natural; his appetite voracious. He is intolerant of heat, and his body very susceptible to the influence of a current of cool air. He complains of no bad taste in his mouth, and his food is keenly relished by him—the taste thereof being natural. It requires a larger dose of an emetic to vomit him than it does in health; there is rarely any nausea about the stomach. A sense of weakness about the small of his back is complained of, and his kidneys perform their duty irregularly. Sometimes he passes large quantities of very clear urine, (hysterical urine), and again, the quantity is small, passed often, and rich in the brick dust sediment. During the latter part of the day, after stirring around sufficiently to raise the circulation above par, and to generate sufficient animal heat, he is tolerably comfortable—is not often called to stool. He brings himself to imagine each night that at last he is to enjoy a "good night's rest." He retires for the night—he easily falls to sleep—his body in a few hours loses most of its animal heat—his pulse falls below par—the fire goes out—he is awakened by an excruciating pain in his bowels—he feels cold and chilly, and an irresistible desire to go to the "water-closet." He hurriedly reaches the closet, (his sphincters are relaxed, and will not wait upon him as of yore); he passes a small, a light, or a brown, or a darker shade of stool, and of a most pungent, offensive character—so pungent as to penetrate a large house, up-stairs and down. This intolerable smell is owing, I think, to the process of fermentation going on in the stomach, instead of digestion. I have never smelt anything so offensive as this chronic diarrhœa discharge. I think this peculiarly offensive discharge pathognomonic of the disease. It is equally offensive to the patient himself as it is to others. After each stool he feels better; returns to bed only again and again every few minutes to jump out and run to the "water-closet." This state of things usually continues until he gets up and stirs around enough to produce some acceleration of his sanguiferous system, and thereby generates some animal heat—or, in other words, until he has kindled his "internal fire." This generally takes until ten or eleven o'clock. From the time his internal combustion gets under way until he goes to bed again at night, he feels tolerably comfortable. But each night, for weeks, months, years, is but a repetition of the one I have attempted to describe above—until, the patient worn out at last, death comes to his relief. He dies, because this "apparatus of combustion" from some cause could not properly burn the fuel put into it—or, in other words, his stomach could not, or rather did not, properly digest the food carried into it.

Of all the causes enumerated in their long catalogue, by Drs. Watson and Condie, as capable of producing diarrhœa, the "mental emotions," in all probability, are the only ones exerting any direct influence in the production of "epidemic chronic diarrhœa," either in its endemic, epidemic or sporadic form. Anger, joy, grief and fear were mental emotions very common indeed; these emotions were almost daily exercised by a very large proportion of our people during the late war. High living and debauch could have manifested but slight power in provoking this disease, for it is a notorious fact that in 1863 our people were subsisting on "half rations," and were, moreover, totally deprived of all the luxuries of life, and almost all were drinking rye coffee and eating cold wheat bread. I will mention another singular fact. It is this: Whilst many old cases of indigestion and dyspepsia were cured by this plain, scanty, frugal diet, other previously healthy persons were becoming affected with chronic diarrhœa. This I can explain on the supposition that these "mental emotions" were stimulating the "nerve centres" of the healthy to such an extent that it resulted in irritation to them, and this irritation was shown by the occurrence (with some epidemic influence prevailing in our atmosphere) of "epidemic chronic diarrhœa." The "nerve centres" of the old dyspeptic were, on the other hand, improved by these "mental emotions," the stimulation proving to them a benefit rather than an injury; they were roused from their torpid, lethargic state, and the functions of the stomach were the better performed—as digestion improved, their dyspepsia was cured. To cure a recent sore, I would apply cooling, soothing applications. To an old, indolent sore, to effect a cure, I always apply stimulating applications. "The season" could exert no baneful influence, for we will find this disease more prevalent in the cold, damp spring and winter months, than in the dry, hot months of summer. But the greatest difference in the two diseases is to be found in the fact that in the "epidemic chronic diarrhœa" the attack commences, or at least is much worse, in the night, when the patient is quiet in bed, when his pulse has reached its lowest standard, and his body has parted with the largest amount of animal heat.

Again, this form of diarrhœa may be said to be benefited by a certain amount of physical as well as mental labor. Common chronic diarrhœa, on the other hand, commences or is worse in the day-time, when the pulse is at its maximum, the animal heat of the body is up to its full standard, and is rendered worse by the attempt upon the part of the patient to undergo the least mental or physical exercise, and is always somewhat improved by the strict observance of quiet, with an abandonment of all mental or physical labor. Then I ask the question, Is it reasonable to suppose that two diseases, differing the one so materially from the other, not only in its inception, but also in its semiology, pathology, etc., should be subject to the same plan of treatment with any hope of cure? Is it not more in accordance with the dictates of a sound, discretionary judgment to believe that, before there is any reasonable expectation of a cure being effected, they should be subjected to plans of treatment differing the one from the other radically? And this, I do not hesitate to affirm, will be found by experience to be necessary, before there is a reasonable hope of curing these two diarrhœas, the one differing from the other "*toto celo.*"

For the sake of simplifying the treatment of "epidemic chronic diarrhœa," I am in the habit of dividing it into four varieties of the same species. And first, those cases arising simply from the want of "animal warmth;" secondly, those cases arising from some irritation about the "nerve centres;" thirdly, cases arising from the same cause, but terminating in acetic fermentation; fourthly, those cases terminating in putrid fermentation. The acetic is recognized by a continued "*rumbling*" in the small intestines; the putrid fermentation by a greater *rumbling*, and in the larger intestines, and which takes place during the act of defecation.

To prove that the food passes through the stomach and through the bowels just as it was swallowed, you have only to make the patient pass his "stools" in a fine wire gauze, and subject the dejecta so caught to repeated washings, when the food can be proven to have passed just as it was swallowed, receiving little or no change from the solvent action of the "gastric juices." Many, very many, cases were diagnosed by this plan. But I will now place upon record only a few well-remembered cases of each variety of this disease, stating my treatment for each variety of the same.

During the war, for four months I suffered terribly from this "chronic diarrhœa," until I was near unto death. I would feel tolerably comfortable during the day, most of the time able to ride about and practice my profession; but I found as soon as I went to bed, I would in a few hours thereafter be compelled to jump up and go to "stool." This had to be repeated every few moments until I got up in the morning and stirred about, whereupon I would become more comfortable and my bowels greatly more quiet. I found the disease invariably came upon me at that hour of the night when my pulse was most below par, and the surface of my body had lost most of its "animal heat," besides its being hastened and aggravated by a scarcity of bed-clothing. Mercurials, chalk mixtures, opiates, antacids and astringents all had been tried, without the least benefit. I now determined to keep my body warm, and my pulse all the time a little above fever heat. With this determination in view, I procured some old, oily apple brandy, distilled from the cider, and I would take a "nip" about every three hours during the day. At night I would place a bountiful supply convenient to my bedside, so I could, whenever I awoke in the night, take a "suck" without getting out of my bed. I at once received marked benefit by this course of treatment; yet, generally just before daybreak I would have to visit the water-closet. My appetite had been very good, indeed, all the while. I now, in addition, determined to eat only two meals in the day—taking my two meals at nine o'clock, A.M., and at four o'clock, P.M.—and my diet to be (avoiding all slops) exclusively of roast-beef, cooked rare, and stale wheat-bread. My improvement was now wonderful, marvelous; I was soon cured, and now, although this disease carried me down to one hundred and twenty, I weigh one hundred and ninety pounds, and can eat late suppers with the next man without any fear of trouble.

My youngest daughter, a sprightly child of ten summers, was for months and months ill with this disease. She was attended by several of our best physicians, with no improvement. Her case was considered hopeless, and had been abandoned by her physicians to Nature. I gave her the brandy, rare roast-beef, and the gravy therefrom poured over well-boiled rice. Her appetite was voracious; she would cry for the brandy—consumed large and enormous quantities of it, by night and by day. She soon recovered, and is now twelve years of age, large, hearty and strong, I venture to assert, as any twelve-year-old to be found in the State of Virginia.

On the 4th day of May, 1867, I was called to see the wife of J. G., a man of color, and, at the time, my blacksmith. I had just come out at the door of his house to start home, when a one-horse wagon drove up. In this wagon lay his sister, N. G., the most pitiable object I ever beheld. He said he had sent for her to die at his own house—that all her physicians had abandoned her case, as one rapidly dying of "phthisis pulmonalis." Without examining into her case at all, I said to him, "From her general appearance, I presume her physicians are correct in their opinion." She was lifted from the wagon and carried into the house, more dead than alive. After a little while he proposed I should go in and examine his sister. This I at first declined to do, from the fact that her appearance was so emaciated and wretched that it was heart-distressing to look upon her. I, however, finally yielded to his urgent request, and rëentered the house for the purpose of examining her. I found her with a pulse too rapid for counting with any certainty (I should say at least one hundred and fifty); her face and body covered with the peculiar cadaverous perspiration; respiration hurried; eye bright and lustrous; the dilatation of the heart very feeble, and synchronous with the beat of the radial artery. She is too sick to speak, and I examine her lungs as she lies in bed the best I can. I am surprised to find her lungs so good. I next examine her stomach; here there seems to be only debility. Then her bowels; from the rumbling and peculiar gum elastic feel of the bowels, I diagnose her case to be chronic diarrhœa from irritation of the great "nerve centres."

℞.—Sulph. quinine, grs. iii.; carb. iron, grs. v.; mixed a powder, to be taken every four hours, if awake; apple brandy *pro re nata;* roast-beef, cut thin and cooked rare; also, a little rice, with the gravy from the rare meat poured over it—to be given a little at a time and often. If alive, I promised to return in two days.

May 6th.—I find the patient apparently about the same, save she can be better raised up in bed; can talk a little; says she has had a "bowel complaint" for four years, and is worse at night. ℞. Strychnia, one-twentieth of a grain; Vallet's carb. ferri, grs. v.; *ter en die,* half an hour before eating; diet, etc., as before.

May 10th.—Is improving finely; perspiration gone; pulse 100; symptoms all measurably improved; slight tenderness over epigastrium; apply fly-blister to epigastrium, and continue as before.

May 14th.—Pulse 85; is setting up, feels well; continue the prescription as before.

May 20th.—Is walking about the house; is beginning to take on flesh; animal heat greatly increased. ℞.—Strychnia, one-sixteenth of a grain; Vallet's carb. ferri, five grains; morning, noon and night, half an hour before eating.

May 30th.—Is well, and has fattened. Continue the prescription for one month longer.

Did not see her after this, but received a message a few days ago by her sister, telling me she was well and hearty, and weighed over two hundred pounds. I do not think she could have weighed over seventy-five pounds the first day I prescribed for her.

A gentleman came to my house to consult me in May, 1865. He was a prominent citizen, a member of the Legislature; said he contracted the disease in Richmond. I was inclined to believe his case to be from acetic fermentation, from the rumbling heard over the region of the duodenum and jejunum. I placed him upon hyposulphite of soda and charcoal; whisky in the place of apple brandy; diet and regimen same as in other cases. He rapidly recovered from his diarrhœa—so I was informed by his son a few days ago.

In putrid fermentation, characterized by more rumbling in the colon and rectum, and by a great escape of sulphureted hydrogen during the act of defecation, you will find the creosote and charcoal to be the best remedy. I should think pepsin another good remedy in these cases; but in all I would not omit the brandy or the whisky, so the heat of the system may always be above fever heat. I think the nerve centres becoming weakened, the gastric juices are thereby impaired and lose their solvent properties measurably, and instead of digestion (or combustion) being performed, we have a process of fermentation setting in. There is nothing more nutritious or more easy of digestion than rare roasted beef. If beef is cooked done, the albumen coagulates and becomes hard and indigestible. But then I am no French cusine, and do no more believe in the doctrine of "alimentation" than I do in homeopathy.

Therefore, I will close this long, rough communication by wishing the SOUTHERN MEDICAL RECORD, its editors, publishers, compositors, contributors and readers a prosperous and a happy New Year.

[NOTE.—One of Virginia's fairest as well as most intelligent daughters, Mrs. W. S. M., informed me yesterday that during the war she was an excruciating sufferer from this disease. Was treated by several eminent physicians of Albemarle county, with mercurials, opiates, antacids and astringents, with no improvement or relief to her sufferings. This highly interesting lady was the fortunate owner of a large, fine pet cat. After months of intense suffering from the turn of the night until ten o'clock next day—at which time she was able to attend to her household duties in tolerable comfort—she awoke one morning surprised to find herself feeling warm and comfortable, and entirely free from pain. Upon examination, she found the warmth and comfort to proceed from the body of her pet cat, lying close to her back. Upon repeated trials she found whenever the cat slept with her she was free from pain, comfortable, and not disturbed by her bowels. I propose to furnish in this disease "animal warmth" to the patient—the *sine qua non*—by my rare beef and "apple brandy," and not by the application of a cat to the back.]

